# Potential Influences of Climate and Nest Structure on Spotted Owl Reproductive Success: A Biophysical Approach

**DOI:** 10.1371/journal.pone.0041498

**Published:** 2012-07-31

**Authors:** Jeremy T. Rockweit, Alan B. Franklin, George S. Bakken, R. J. Gutiérrez

**Affiliations:** 1 Department of Fisheries, Wildlife, and Conservation Biology, University of Minnesota, Saint Paul, Minnesota, United States of America; 2 National Wildlife Research Center, United States Department of Agriculture, Fort Collins, Colorado, United States of America; 3 Department of Biology, Indiana State University, Terre Haute, Indiana, United States of America; University of Regina, Canada

## Abstract

Many bird species do not make their own nests; therefore, selection of existing sites that provide adequate microclimates is critical. This is particularly true for owls in north temperate climates that often nest early in the year when inclement weather is common. Spotted owls use three main types of nest structures, each of which are structurally distinct and may provide varying levels of protection to the eggs or young. We tested the hypothesis that spotted owl nest configuration influences nest microclimate using both experimental and observational data. We used a wind tunnel to estimate the convective heat transfer coefficient (*h_c_*) of eggs in 25 potential nest configurations that mimicked 2 nest types (top-cavity and platform nests), at 3 different wind speeds. We then used the estimates of *h_c_* in a biophysical heat transfer model to estimate how long it would take unattended eggs to cool from incubation temperature (∼36°C) to physiological zero temperature (PZT; ∼26°C) under natural environmental conditions. Our results indicated that the structural configuration of nests influences the cooling time of the eggs inside those nests, and hence, influences the nest microclimate. Estimates of time to PZT ranged from 10.6 minutes to 33.3 minutes. Nest configurations that were most similar to platform nests always had the fastest egg cooling times, suggesting that platform nests were the least protective of those nests we tested. Our field data coupled with our experimental results suggested that nest choice is important for the reproductive success of owls during years of inclement weather or in regions characterized by inclement weather during the nesting season.

## Introduction

The ability of many bird species to build or modify their nests in response to environmental stressors, such as climate, is an important behavior linked to nestling survival and ultimately, reproductive success. Because nestling survival and recruitment affects fitness of parents, the nest building behavior is under tremendous selective pressures [1]. Behavioral responses to environmental stressors such as ambient temperature and wind generally include varying nest placement and modifying nest construction [2]–[9]. For example, several studies have shown that birds breeding in colder regions build thicker nests than conspecifics breeding in milder regions [8]–[10]. Additionally, Reid et al. [11] found that ground-nesting pectoral sandpipers (*Calidris melanotos*) constructed nest scrapes that simultaneously minimized both conductive heat loss to the ground and convective heat loss. Studies have also demonstrated the importance of nest placement with regard to solar radiation (i.e., either increasing radiation in colder regions or decreasing radiation in warmer regions [2], [12], [13]). Burton [13] elucidated a relationship between nest entrance orientation and latitude among ground-nesting passerines. He found a strong trend for preference of north-facing nests at lower latitudes, east-facing nests at mid-latitudes and south-facing nests at upper latitudes and attributed this to the variation in ambient temperatures across this gradient.

However, not all birds build or modify their own nests. Instead, they must select existing nest structures that minimize predation pressures (see [1], [4], [5], [14], [15]) while simultaneously protecting eggs and young against climatic extremes. Because selection of a good nest site is critical for nestling survival, it should be subject to the same selection pressures as nest building behavior is in other birds.

**Figure 1:**
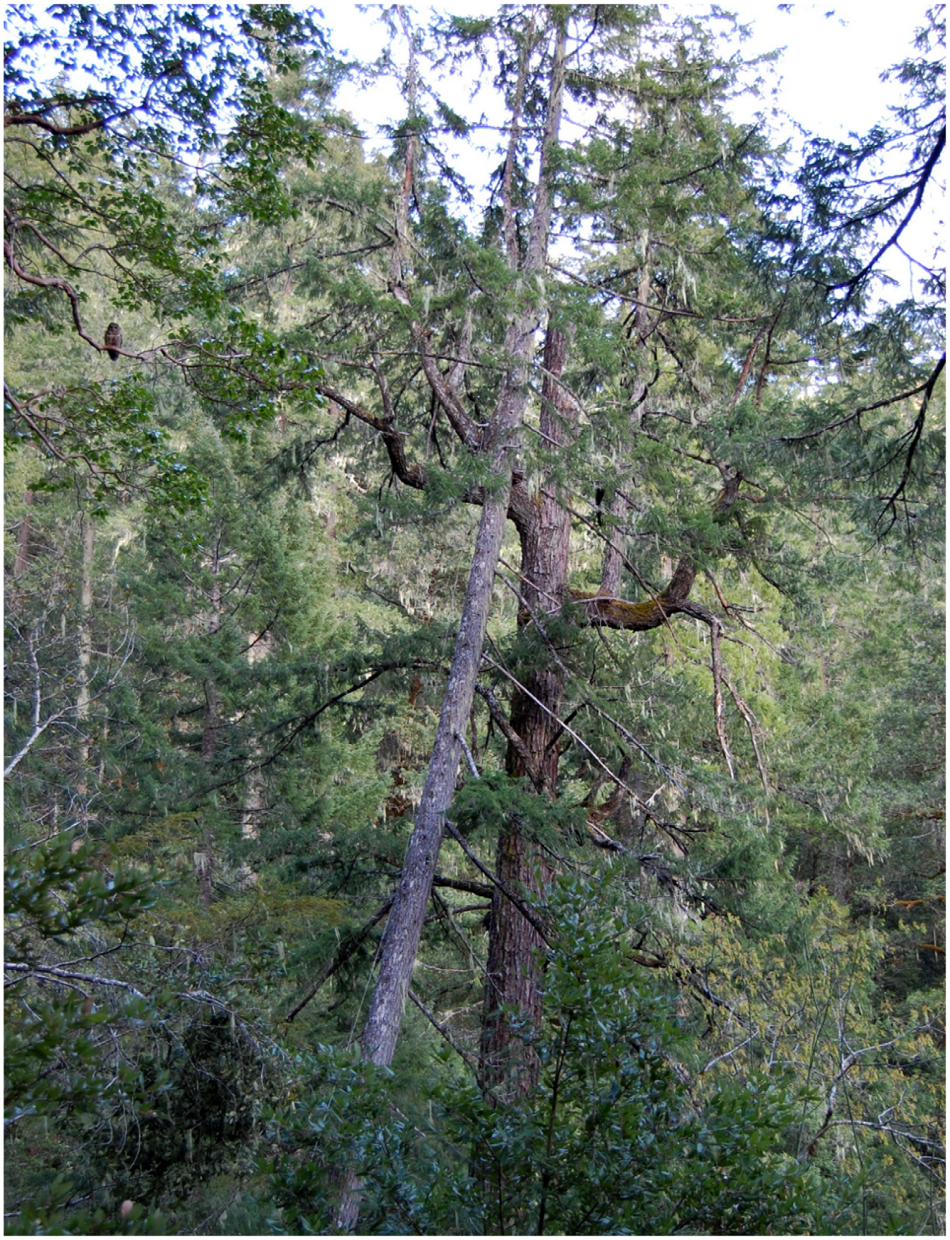
Typical spotted owl top-cavity nest. Follow the bole of the tree upwards to note the broken top chimney. Multiple banner limbs and a nearby tree extend above the chimney. Note the spotted owl perched in an adjacent tree (left-side of image) for scale.

Spotted owls (*Strix occidentalis*) do not build or modify their nests. Instead, they select one of three types of existing nest structures: platform nests, top-cavity nests, or side-cavity nests. Platform nests include debris accumulations, mistletoe (*Arcuethobium* spp.) infections, or abandoned nests of other animals; they are used predominately in coastal and intensively managed forests [16], [17] whereas cavity nests are used more frequently in interior and mature/old growth forests [18], [19]. Top-cavity nests are formed after the bole of a tree breaks off and subsequent decay forms a chimney-like cavity inside the bole of the tree (the term “top-cavity” refers here to this nest type while “chimney” refers to the specific structural component of this nest type). Banner limbs (horizontally growing branches that assume a vertical growth form after the bole breaks) are frequently associated with this nest type (Fig. 1). Side-cavity nests are usually formed when a limb breaks from the bole of a tree followed by decay at the site. Each nest type has different structural properties which may provide different levels of protection to the eggs, young, and incubating female.

Spotted owls begin nesting in early spring [20] when winter-like conditions (i.e., cool, rainy weather) may persist into the nesting season. Inclement weather during the nesting season has been correlated with low reproduction in spotted owls [21], [22], but the mechanisms responsible are unknown. However, Hirons [23] found that the foraging success of tawny owls (*Strix aluco*) decreased during periods of rainy weather, and speculated the background noise of rainfall impeded their ability to detect prey. During incubation and early brood rearing, male spotted owls provide females with food; females continuously incubate with only brief departures for personal maintenance [24]. If males cannot provision females with sufficient food during periods of extended precipitation, females may be forced to leave the nest to forage, which will increase the vulnerability of eggs or young to thermal cooling. Egg chilling can reduce offspring fitness or cause mortality of eggs [25]–[27]. In addition, spotted owl hatchlings are altricial [20], [28] and rely on the brooding female to aid in thermoregulation [29]. Consequently, selecting a nest site that provides a favorable microclimate which protects eggs or young from cooling when females are absent may be an important factor in egg viability, hatchling growth rates [30] and ultimately reproductive success.

Field observations suggest that spotted owls may prefer certain nests. Some nests within a territory are used more often than others, even when nests do not appear to be a limiting factor [24], [31]. Similarly, North et al. [31] found that nests used repeatedly by California spotted owls (*S. o. occidentalis*) had more than twice the weighted reproductive rate of owls using a nest only once, regardless of the different individuals using those nests. Thus, we hypothesized that structural differences among nest types create different thermal conditions within the nest, which could influence reproductive success during inclement weather, but not during mild weather. We predicted that deep top-cavity nests would be more protective than shallow top-cavity nests, and that top-cavity nests would be more protective than platform nests. We did not evaluate side-cavity nests because they are relatively rare in our study area [32].

To test this hypothesis, we first estimated parameters needed for a biophysical heat transfer model to evaluate the relative cooling rates of unattended eggs within nests of differing configurations. We then used weather data from a Remote Automated Weather Station (RAWS) near our study area as input to this model to estimate how nest configuration might affect the cooling rates of eggs under both mild and inclement weather conditions. Finally, we interpreted these results in the context of the nesting, brooding, and foraging behavior of northern spotted owls.

## Materials and Methods

### Field Measurements

We conducted our study in northwestern California, Humboldt and Trinity County, USA as part of a long-term (1985–2011) demographic study of northern spotted owls (*S. o. caurina*; [22]) under the guidelines of Colorado State University institutional animal care and use protocols (05-006A and 08-011A), federal threatened species permit (TE026280-12) federal banding permit (21350) and California state permits (SC-880 and SC-005219). Topography on the study area was mountainous, with elevations ranging from 150 to 1700 m. The dominant vegetation was mixed evergreen forest composed predominately of a Douglas fir (*Pseudotsuga menziesii*) overstory and a hardwood understory of tanoak (*Lithocarpus densiflorus*), madrone (*Arbutus menziesii*) and canyon live oak (*Quercus chrysolepis*). Climate on the study area was Mediterranean with cold, wet winters and hot, dry summers [33].

To provide input data for the heat transfer model, we used weather conditions that owls experienced during April, the early part of the nesting season. We obtained temperature and wind speed data from nearby Remote Automated Weather Stations (RAWS) operated by the Western Regional Climate Center, Desert Research Institute, Reno, Nevada (http://www.raws.dri.edu/wraws/ncaF.html). Wind speeds at the RAWS were recorded at about 6 m above ground level, while mean nest height was >17 m. Mean wind speeds from RAWS likely underestimated actual wind speeds at nest height because wind speed increases with height above the ground and topographical differences influence wind speeds [34], [35]. Therefore, we used maximum recorded wind speeds because we felt they would be more representative of wind conditions at the nest.

We believed forced convection (i.e., wind) would be the predominant mode of heat transfer in the thermal energy budget of eggs that would be most influenced by nest configuration. The spatial complexity of convection requires empirical measurement of convective heat transfer coefficients (*h_c_*). Because real nests are often difficult to access in the field and too large to be brought into wind tunnels, we used a scale model of a nest tree and adjusted the results to full-sized trees using dimensional analysis. We measured the structural characteristics of real nests used in our study area and then used these measurements to design a scale model top-cavity nest.

Spotted owls occupy top-cavity nests that are successful or unsuccessful with respect to the reproductive outcome of birds using those nests. We defined successful nests as top-cavity nests where owls successfully fledged young during years with overall low reproduction within the study population, based on annual mean reproductive output relative to the 22-year norm. We categorized the years 1993, 1995, 1999 and 2003 as years of low reproduction (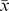 = 0.164 fledge/pair, SE = 0.041), and the remainder as years of average reproduction (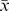 = 0.536 fledge/pair, SE = 0.048). Years of low reproduction also had higher than average precipitation, which has been correlated with lower reproductive output in both spotted and tawny owls [22], [23].

Because owls using successful nests reared young in low reproduction years, we suspected they had structural features that made them superior to other top-cavity nests in our study area. From the set of owls using these “superior” nests, we examined their reproductive histories to ensure that our classification of nests was not confounded with owl pair quality (see [36] for details). We climbed and measured structural characteristics we thought might affect the microclimate of the nest. These were: 1) tree height, 2) nest height, 3) diameter at breast height (dbh), 4) nest diameter (taken as the average of two perpendicular measurements of inside nest diameter), 5) height of continuous chimney (taken from the nest floor to the highest point where a chimney is completely enclosed), 6) height of total chimney (taken from the nest floor to the highest point of the chimney wall), 7) azimuth of chimney split, if present (a chimney split is a vertically oriented opening in the chimney wall that results in the loss of a portion of the chimney wall), 8) number of banner limbs (taken as the number of vertical limbs that extended above the top of the chimney and were >10 cm in diameter), 9) azimuth of banner limb(s), 10) diameter of banner limb(s) (at nest height), and 11) distance of banner limb(s) to chimney. A banner limb is a limb that becomes the new dominant terminal leader and assumes a vertical growth form after the original terminal leader is broken off. We averaged nest diameter, banner limb diameter, and distance of banner limb to chimney of the “superior” nests and used these dimensions as metrics to scale our model nest. We calculated Rayleigh test statistics using program Oriana (Kovach Computing Services, Anglesey, Wales, U.K.) to determine if there was any directedness in banner limb azimuth or azimuth of chimney split.

### Experimental (Wind Tunnel) Measurements

**Figure 2:**
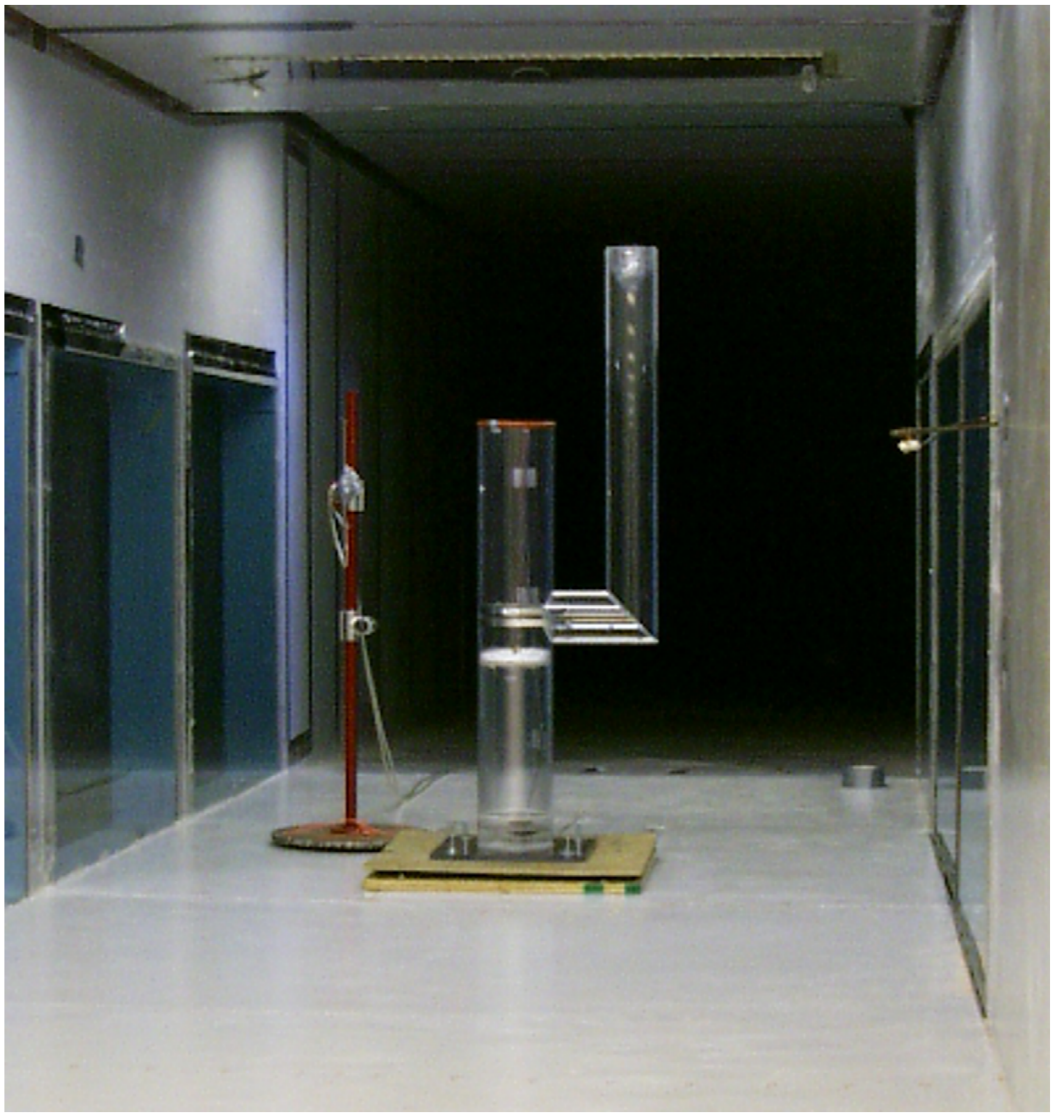
Scale model nest tree setup we used to calculate the *h_c_* of unattended eggs.

Both the model nest and model eggs were scaled to a ratio of 1∶3.6 to ensure the geometric similarity required to apply results from our model nest to full-sized nests. We constructed our model nest chimney of acrylic tubing having a 15 cm inside diameter (Fig. 2). We added an internal platform to support the eggs that could also be adjusted to vary chimney depth. Chimney depth was the vertical distance from the internal platform to the top of the chimney. The adjustable chimney depth of our model nest allowed us to simulate platform nests because they could be defined simply as a top-cavity nest with a chimney depth of zero. We attached a single 10.3 cm outside diameter model banner limb without branches or needles 33.5 cm below the chimney opening such that it extended 34.5 cm above the opening. The entire model could be rotated to simulate different banner limb positions relative to the wind.

To simulate owl eggs, we used two 12.7 mm diameter, gold-plated, solid bronze spheres (hereafter referred to as “model eggs,”). Gold plating minimizes heat transfer by thermal radiation and facilitates more accurate measurement of convective heat exchange. These model eggs corresponded to spotted owl egg diameters of 46.5 mm (average of the polar and equatorial diameters). While real spotted owl eggs are prolate spheroids, the differences in volume and surface area between real eggs and our model eggs was only 9.4% and 8.5%, respectively. Thus, the spherical model eggs closely approximated spotted owl eggs. We drilled a 1 mm diameter hole through the model eggs, and then counter-drilled a 3.2 mm diameter hole approximately ¾ of the way through the sphere. We placed a 900 ohm 0.1% precision metal-film resistor in the larger hole to heat the model, and placed the junction of a type-T thermocouple wire in the smaller hole. Thus, the thermocouple wire measured model egg temperature near the edge of the sphere, while the resistor was located near the center.

We conducted all experiments using the boundary layer wind tunnel at the University of Minnesota’s St. Anthony Falls Laboratory. This wind tunnel had a 1.7 m×1.8 m working section and a maximum operating velocity of 45 m/s. We placed our model nest containing 2 model eggs inside the working section (Fig. 2). We modeled various nest structures by varying the chimney depth and banner limb position to estimate the effect of such variations on the rate of convective heat loss of eggs inside the nest. We tested model chimney depths of 0 cm, 7.5 cm, 15 cm, 30 cm, and 45 cm, resulting in dimensionless nest depth to nest diameter ratios (NDD ratio) of 0, 0.5, 1, 2, and 3, respectively. We tested the effect of a banner limb on convection by making measurements without the model limb and with it while placed at 0°, 60°, 120°, and 180° azimuths relative to wind direction (0°). We assumed that results with the simulated limb at 300° and 240° would be the same as those at 60° and 120°.

We measured air temperature (°C) inside the wind tunnel with a type-T thermocouple and wind speed (m/s) with a pitot tube placed adjacent to the model nest to avoid boundary layer interactions. We recorded 1-minute averages of wind speed, air temperature, egg temperature, and the electrical power supplied to the resistor to heat the egg using a Campbell Scientific CR5000 data logger (Campbell Scientific, Inc., Logan, UT). We used 25 nest configurations and 3 wind speeds (7.7 m/s, 15.8 m/s, and 23.5 m/s) resulting in 75 combinations. We supplied constant heater power to the eggs and tested each combination for 12 minutes. The first and last minute of each test was eliminated to minimize the effect of irregularities caused by starting and stopping each test. We randomly selected 15 combinations for replication (9 were replicated twice, 6 were replicated once). Because the temperature of the model eggs approached steady-state values exponentially, we extrapolated our data to find the final equilibrium temperatures using program NLREG [37]. Joule heat generated by the wind turbine caused air temperature to change during the experiment, and was extrapolated similarly. The average R^2^ value for these extrapolations was 0.991 (95% CI = 0.988, 0.995).

### Heat transfer analysis

The steady-state heat transfer equation for both real and model eggs is: 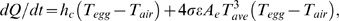
(1) where *dQ/dt* [W] is the rate of heat gain or loss *Q* per unit time *t*, *T_egg_* [K] is egg temperature, *T_air_* [K] is air temperature in the wind tunnel, *T_ave_* [K] is the average temperature of the eggs and the air and 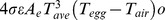 [W] is the linearized form of the Stefan-Boltzmann equation [38], with 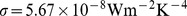, *ε*  =  thermal emittance [dimensionless, ranges from 0–1], and *A_e_*  =  the area of the egg emitting thermal radiation. We calculated *h_c_* by rearranging the equation as:



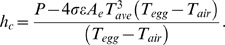
(2)Because gold plating the model egg reduces ε to about 0.05, nearly all of the electrical heater power 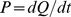 is lost by convection which facilitates accurate measurement of *h_c_*. We calculated *h_c_* for each egg separately and then averaged these to compute one value of *h_c_* for each nest configuration.

The convection coefficients of our scale model eggs within a scale model nest can be accurately applied to real spotted owl eggs in a full-sized nest by using dimensional analysis [39]. Briefly, dimensionless numbers are generalized representations of complex processes. For convection, these are the Nusselt Number, Nu (heat transfer), Reynolds Number, Re (fluid flow patterns), and the Prandtl Number, Pr (fluid properties) [39]. Any empirical relationship such as 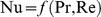 is dimensionally correct. For properly chosen dimensionless numbers, the relation is also physically correct as long as there is geometric-similarity between the model and its subject. As the fluid is air in both the lab and the field, Pr is constant, thus for each nest geometry we only needed an empirical relation between Nu and Re, the size of real and model eggs, and laboratory and field wind speeds to estimate the convection coefficients for real eggs.

Therefore, to relate our laboratory data to actual field conditions, we first computed 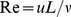 and 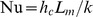 for each model nest configuration and wind speed *u* [m/s] using the diameter of the model egg as the characteristic dimension *L_m_* [m], and the thermal conductivity *k* and kinematic viscosity *ν* of air obtained from standard tables [34]. We then defined empirical Nu-Re relationships for each nest configuration.

We based our analysis and conclusions on the time required for a spotted owl egg to cool from incubation temperature (∼ 36°C) to physiological zero temperature (PZT; ∼ 26°C) following the hypothetical departure of an incubating female. We referred to this as “time to PZT.” The PZT of an egg is the temperature at which an egg’s development stops [25], [27], and hence, has adaptive significance because hatching success may decrease if egg temperature reaches or falls below PZT [27]. Avian species appear to recognize this limitation; for example, 14 passerine species did not let their eggs drop below PZT during incubation [40].

To determine how long it would take an unattended egg to cool to PZT, we evaluated the thermal energy budget equation (1) for specific nest configurations, wind speeds and air temperatures. We computed Re for each field wind speed using L_e_ = 0.0465 m for owl eggs. We then found Nu from the empirical Re-Nu relationship determined in our wind tunnel study, and then computed the convection coefficient as 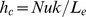. For a cooling egg, 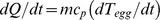. Substituting into equation (1) and rearranging yields:
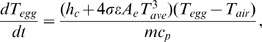
(3)where *m* is the fresh mass of a spotted owl egg, *c_p_* is the specific heat of an egg assumed to be 3.7 Jg^−1^°C^−1^ [41], *T_egg_* is egg temperature, and *T_air_* is air temperature. We assumed the thermal emissivity of owl eggs 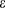 *= *0.98 (typical of rough natural surfaces; [39]). We estimated egg mass as *m* = 50 g [36] from the dimensions of a northern spotted owl egg because there were no data for this species.

The time to PZT for a given nest configuration and environmental condition was calculated by using Simpson’s rule to integrate equation (3) until *T_egg_* =  PZT (26°C) and recording the elapsed time. We calculated time to PZT for each nest configuration during mild weather (*T_a_* = 9°C, *u* = 1 m/s) and during inclement weather experienced during an early nesting season storm (*T_a_* = −2°C, *u* = 10 m/s). The absolute difference in time to PZT between the two different weather scenarios for a given nest configuration defined the weather effect for that nest configuration.

We also quantified the effect of nest configuration on time to PZT by calculating time to PZT for most protective nest configuration (that yielding the smallest *h_c_*) and the least protective nest configuration (that yielding the largest *h_c_*) experiencing the same weather conditions (mild or inclement as defined above). The difference in time to PZT between the two configurations was considered the nest effect for that weather scenario.

### Statistical Analysis

We developed a set of *a priori* models that hypothesized relationships between the Nu of the model egg, the structural configurations of the model tree, and Re (Table 1). We used Nu and Re (instead of *h_c_* and wind speed, respectively) because using dimensionless numbers allowed us to apply the results of the wind tunnel simulations to the real nest environment. We then ranked the models using a bias-corrected version of Akaike’s Information Criterion (AIC_c_; [42]). Once models were ranked, we used Akaike weights (*w_i_*) to estimate the relative likelihood that each model best explained the data, relative to the other models we examined [42].

**Table 1:** *A priori* hypotheses examining the influences of nest structure on the Nu of the eggs.

Hypothesis	Model structure
**θ_DEPTH+RE_**	
Nu decreases as depth increases, and increases as Re increases.	*β_0_ + β_1_DEPTH + β_2_RE*
**θ_DEPTH+LIMB+RE_**	
Nu decreases as depth increases, and decreases in the presence of a banner limb. Banner limbazimuth affects Nu. Nu increases with Re.	*β_0_ + β_1_DEPTH + β_2_BL + β_3_RE*
Nu decreases as depth increases, and decreases in the presence of a banner limb. Banner limbazimuth affects Nu. Nu increases with Re, and the effect of banner limb decreases as depth increases.	*β_0_ + β_1_DEPTH + β_2_BL + β_3_RE + β_4_DEPTH*×*BL*
Nu decreases as depth increases, and decreases in the presence of a banner limb. Banner limbazimuth affects Nu. As Re increases, Nu increases and the effect of banner limb increases.	*β_0_ + β_1_DEPTH + β_2_BL + β_3_RE + β_4_BL×RE*
Nu decreases as depth increases, and decreases in the presence of a banner limb. Banner limbazimuth affects Nu. Nu increases with Re, and the effect of banner limb decreases as depth increases.The effect of banner limb increases as Re increases.	*β_0_ + β_1_DEPTH + β_2_BL + β_3_RE + β_4_DEPTH×BL + β_5_BL×RE*

Nest structures examined included chimney depth (DEPTH), banner limb position (BL) and Reynold’s number (RE). RE and DEPTH were modeled in linear and log-linear form.

Our response variable was the natural log (log_e_) of Nu of the eggs to insure normality in our data, and our explanatory variables were chimney depth, banner limb position, and Re. Chimney depth and Re were modeled as continuous variables. Because of the complexity of wind flow patterns [34], we did not think it was appropriate to model banner limb as a continuous variable, and thus modeled it as a categorical variable. Chimney depth and Re were examined in linear and log-linear forms to estimate their relationship with Nu. All analyses were performed using the GENMOD procedure in program SAS (SAS Institute Inc., Cary, NC 2004).

## Results

Eleven of 168 known top-cavity nests fit our definition of a “superior” nest and we climbed and measured 7 of these. We did not climb the 4 remaining nest trees because of safety concerns (i.e., nest trees were decayed and unstable). We only measured “superior” nests in this study; therefore these measurements are only useful for describing characteristics of these nests and do not imply any differences with other nests in the study area.

Nest tree measurements such as tree and nest height, and nest tree DBH agreed closely with other studies (Table 2; [19]). Based on coefficients of variation, these measurements, along with inside nest diameter and banner limb DBH, did not vary much among nests. The orientation of banner limb azimuth was random (*r* 0.14, *n* = 14, *p* = 0.78), as was azimuth of chimney split (*r* = 0.29, *n* = 8, *p* = 0.53). The largest easurement of continuous chimney depth (165 cm) was used as the maximum chimney depth of the model nest (45 cm when scaled to the model nest). The mean NDD ratio for “good” nests varied greatly depending on which chimney depth measurement was used (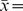
0.9; 95% CI = 0.04**–**1.76, for continuous chimney depth, versus 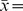
5.6; 95% CI = 3.22**–**7.98, for total chimney depth). This discrepancy was the result of 3 nest trees with continuous chimney depths = 0 because of a split in the chimney that extended to the bottom of the nest platform.

**Table 2:** Descriptive statistics of structural and nest tree characteristics for successful spotted owl nest trees (*n* = 7) measured in the field.

Nest Characteristic	Mean	StandardDeviation	Coefficientof Variation
Tree height	47.6 m	5.9	12.5
Nest height	27.3 m	4.7	17.3
Nest tree DBH	132.5 cm	21.9	16.5
Inside nest diameter	52.9 cm	13.9	26.2
Continuous chimney depth	47.1 cm	62.8	133.2
Total chimney depth	350.1 cm	219.1	62.6
Azimuth of chimney split	155°	90°	–
Banner limb azimuth	327°	114°	–
Banner limb DBH	41.3 cm	12.7	30.8
Distance to banner limb	87.3 cm	113.4	129.8
Nest depth to diameter ratio†	5.6	3.0	53.6

† Nest depth: diameter ratio was calculated using total chimney depth.

### Effect of Nest Configuration on the Nu of Unattended Eggs

We examined 16 *a priori* hypothesized models that described different relationships between nest configuration, Re, and the Nu of the egg (Table 1). Based on Akaike weights, our top-ranked model (Model 1, Table S1) was nearly 3 times more likely, given the data, than the second-ranked model, indicating this model performed better than our other models.

Because we wanted to predict values of Nu, and ultimately *h_c_*, from other wind speeds not tested in the wind tunnel, we added a *post hoc* model based on the top-ranked model (Model 1, Table S2) by 1) removing two banner limb by Re interactions terms that had uninformative 95% confidence intervals that overlapped zero, and 2) collapsing two chimney depth by banner limb interactions terms into a single term because they had nearly identical parameter estimates. Applying these *post hoc* procedures to the top *a priori* model did not alter our inferences, but it did increase the precision of the remaining parameters.

**Figure 3:**
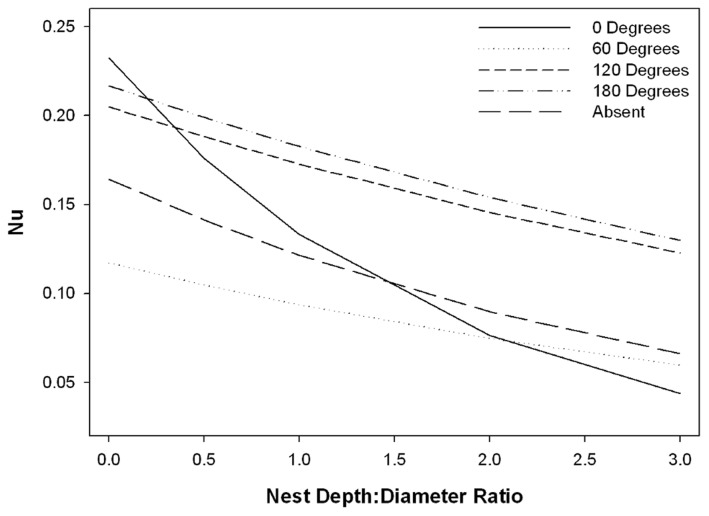
Effect of banner limb position and NDD ratio on the Nusselt number (Nu) of eggs. NDD ratio is the dimensionless form of chimney depth. This particular graph illustrates the relationship for a Reynolds number (Re) consistent with an environmental wind speed of 2.3 m/s, but this general relationship held for all wind speeds tested. Because we minimized other forms of heat loss, Nu roughly corresponds to the inverse of time to PZT for all wind speeds >1 m/s.

The *post hoc* model became the best approximating model when ranked with the top *a priori* models and based on Akaike weights fit the data better (Table S2). The *post hoc* model indicated that the Nu of the eggs decreased as chimney depth increased (Fig. 3), and increased with Re (Table S3). The effect of banner limb position on Nu was variable (Table S3, Fig. 3). Compared to nests with no banner limb, a banner limb positioned at either 120° or 180° relative to wind direction increased Nu approximately 30%, while a banner limb positioned at 60° decreased Nu approximately 30% (Fig. 3). A banner limb positioned at 0° caused strong turbulent boundary layer interactions between the banner limb and the nest chimney that caused Nu to decrease rapidly as chimney depth increased (Fig. 3). This model also indicated strong interactions between chimney depth and banner limb position.

### Effect of Nest Structure and weather on time to PZT

Time to PZT for all nest configurations during inclement weather conditions (T_a_ = −2°C and *u* = 10 m/s) ranged from 10.6 minutes to 18.7 minutes (>75% difference), while estimates of time to PZT during mild weather (T_a_ = 9°C and *u* = 1 m/s) ranged from 29.2 minutes to 33.3 minutes (∼10% difference).

Two nest configurations consistently had the shortest times to PZT for the range of weather conditions used (i.e., the least protective nests). Both configurations had chimney depths of 0 (NDD ratio = 0), but had different banner limb positions (0° or 180°). In contrast, only one nest configuration had the longest time to PZT for all simulated weather conditions (i.e., the most protective nest). This configuration had a NDD ratio = 3 and a banner limb in front of the nest, relative to wind (banner limb position = 0°). In general, time to PZT increased with increasing NDD ratios, indicating that nests with deep chimney were more protective than nests with shallow chimneys.

Estimates of time to PZT were generally shorter when a banner limb was present compared to when no banner limb was present. Excluding nests with shallow chimneys (NDD ratio ≤1), nests that had a banner limb positioned in front of oncoming wind (banner limb position = 0°) had the longest values of time to PZT. As chimney depth decreased, a banner limb at 60° became increasingly important for lengthening time to PZT, illustrating the interaction between a banner limb at 60° and chimney depth (Fig. 3). A banner limb behind the nest (banner limb position = 180°) resulted in the shortest estimates of time to PZT for all chimney depths tested, except when chimney depth was 0, in which case a banner limb at 0° had the shortest time to PZT.

**Figure 4:**
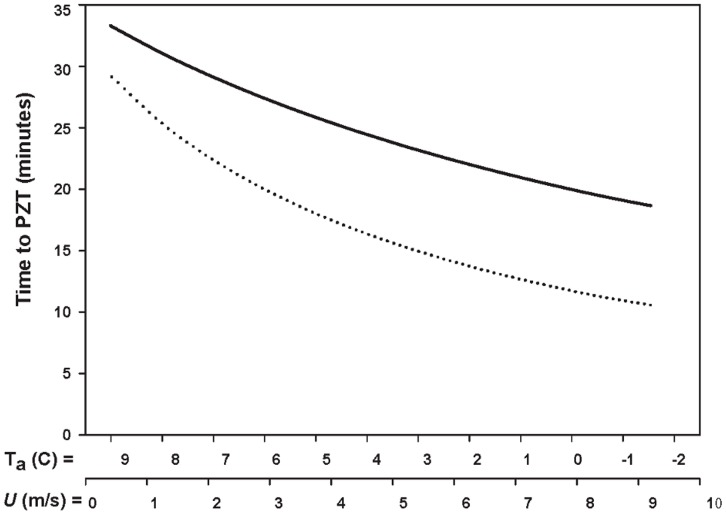
Times to PZT for two nests as weather conditions change from mild to inclement. Estimates are for the most-protective top-cavity nest (solid line) and the least-protective platform nest (dashed line) as weather conditions changed from mild to inclement. Estimates of time to PZT are similar during mild weather, but diverge as weather conditions become more inclement.

As simulated weather conditions changed from mild to inclement, time to PZT for the most protective top-cavity nest went from 33.3 minutes to 18.7 minutes. Under the same simulated weather changes, time to PZT for the least protective platform nest went from 29.2 minutes to 10.6 minutes (Fig. 4). When comparing estimates of time to PZT for the full range of weather conditions tested, the most protective top-cavity nest resulted in times to PZT that were approximately 50% greater than those for the least protective platform nest. Put another way, time to PZT for the most protective top-cavity nest was nearly twice as long as time to PZT for the least protective platform nest during cold, windy conditions, but only 10% different during mild weather (Fig. 4).

**Figure 5:**
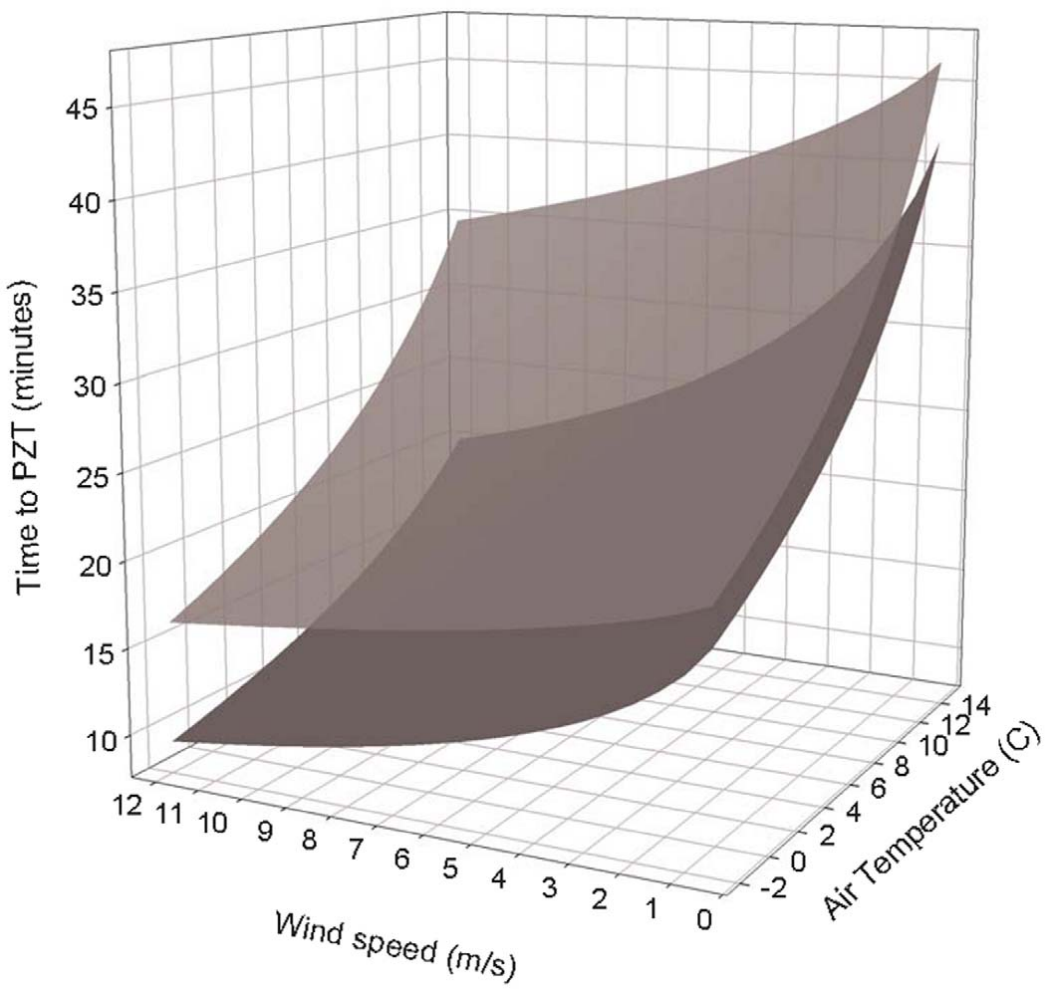
Estimates of Time to PZT for two simulated nests experiencing the same weather conditions. Comparison of times to PZT for one of the superior nests (upper plane) measured in the field and a simulated platform nest (lower plane) for a range of weather conditions experienced by nesting spotted owls in northwest California.

We estimated times to PZT for one of the superior nests and a simulated platform nest experiencing a range of simulated weather conditions (Fig. 5). On average, the superior nest had estimates of time to PZT that were 50% longer than those of the simulated platform nest. Wind speed appeared to be more important in affecting time to PZT than air temperature. During calm conditions, nest structure had little effect on time to PZT, but as wind speed increased; nest structure became increasingly important for protecting the nest contents (Fig. 5).

## Discussion

### Patterns of Nest Type Use

Our results demonstrated that different spotted owl nest structures influence the cooling time of unattended eggs and hence, nest microclimate. We found that platform nests (NDD ratio = 0) consistently had the shortest times to PZT, indicating that these configurations provided the least amount of protection against convective heat loss. Thus, we concluded that top-cavity nests provide a more favorable microclimate than platform nests. However, this distinction was only apparent during simulated inclement weather (i.e., cool and windy; Fig. 4). During mild weather, differences due to nest configuration were small and likely negligible in terms of spotted owl reproductive success. In light of these findings, and the fact that spotted owls nest in a range of climates, we concluded that spotted owls should select nests based on the degree that severity varies within nesting season climates. That is, spotted owls should select a larger proportion of cavity-type nests in regions where the nesting season climate is characterized by frequent, inclement weather events, but in regions with a less variable, mild nesting climate, nest type selection should be less constrained by climate and may more closely reflect availability or some other constraint.

Observations of nest use across their range suggest that spotted owls may indeed be selecting nests based on the nesting season climate of the area. However, spotted owls are also observed using nest types according to land use and fire history. For example, in areas of managed forests where logging has removed the majority of mature trees capable of providing adequate cavities for nesting, spotted owls nest predominately in platform type nests [16], [17]. These patterns have led to two competing hypotheses to explain nest type use patterns of spotted owls [18], [20]. The availability hypothesis posits that nest type use is a function of the availability of such sites and has been tested indirectly by examining reproductive output as a function of nest types, with no relationship found between reproductive output and nest type [18], [19], [32]. However, variation due to nest type may have been minimal if these studies occurred under relatively mild nesting season weather, as suggested by our study (Fig. 4). Selection of a nest type does not necessarily imply preference. Preference can only be inferred when the full complement of nest structures is available and spotted owls nesting in areas lacking mature trees with suitable cavities are limited to selecting among available platform nests.

The climate hypothesis posits that spotted owls select nest types in response to the severity of nesting-season climate of the region. Owls inhabiting areas that experience more severe weather during the nesting season use a greater proportion of cavity-type nests than owls inhabiting areas with more benign nesting weather (e.g., northern vs. southern California; [22], [32]). Presumably, owls use more cavity-type nests in areas having more inclement nesting season weather because they provide more protection for eggs and incubating females. However, climate is likely only one factor spotted owls must consider when choosing a nest site. Other factors such as predation risk, parasite load, and prey availability must be taken into account and the relative importance of these factors likely changes across the species’ range.

### Effect of Structural Configuration on Time to PZT

Both NDD and banner limb position significantly affected time to PZT. In general, a banner limb actually increased the amount of convection from the eggs, thus reducing time to PZT. However, foliage from banner limbs may provide additional overhead protection from precipitation, or radiative heat loss. Spotted owls had higher reproductive success when using nest sites that had an above-nest foliage volume >4300 m^3/^0.05 ha [31]. Precipitation that wets the eggs could reduce hatching success because of a reduction in eggshell conductance of O_2_ and CO_2_ [25], or increased heat loss due to the effects of latent heat loss [35], [39]. Similarly, Bakken et al. [43] found that wetting the down of newly hatched chicks dramatically increased the thermal conductance of the chicks by more than an order of magnitude, which could be detrimental to altricial spotted owl nestlings [28]. Finally, many of the top-cavity nests we examined had more than one banner limb. Although we did not test the effects of multiple banner limbs or foliage, additional banner limbs and foliage would likely increase the amount of overhead protection for a nest.

The influence that banner limb position had on *h_c_* was large, especially when the banner limb was either in front of, or behind the chimney, relative to oncoming wind. These large effects were most likely due to patterns of wind flow around the banner limb [34]. As air flows around a cylinder (e.g., banner limb), it creates a high pressure “bow wave” immediately in front of the cylinder and a region of low-velocity vortices behind the cylinder [34]. Therefore, banner limb orientation may result in either a “bow wave” forcing air down into the chimney (banner limb at 180°), facilitating convective heat loss, or in low-velocity vortices that limit air penetration into the chimney (banner limb at 0°; Fig. 3).

One assumption in our calculations of time to PZT is that radiative heat loss is equal for all nests. This assumption should be valid for nests with deep chimneys (NDD ratios ≥1), but eggs inside nests with shallow chimneys will likely lose more heat to radiation because of increased exposure to the sky which is colder than air temperature [39] and because there is less chimney structure (which is at air temperature) to radiate heat back to the eggs [44]. As a result, differences in time to PZT between natural chimney nests and platform nests will likely be even larger than we report here for our experimental nest configurations.

### Functional Consequences of Nest Type Selection

One potential benefit of selecting a nest with a superior configuration would be an increase in the total area accessible for foraging because spotted owls are central place foragers [45]–[47]. Assuming equal flight speeds and a circular foraging area, the total area accessible to foraging is proportional to the square of foraging time, which for a nesting female spotted owl should be equal to time to PZT of the unattended eggs. Using estimates of time to PZT for a platform and top-cavity nest during inclement nesting-season weather, a female using a platform nest could allocate 11 minutes to forage over 121 units of foraging area. However, a female using a top-cavity nest could allocate 19 minutes to forage over 361 units of foraging area, nearly tripling the area accessible for foraging, assuming equal effort for search time during foraging.

Additionally, if we assume the estimated times to PZT were measures of the insulative quality of the nest, then platform nests were less insulative than top-cavity nests. During inclement nesting-season weather, the eggs inside a nest with a deep chimney (NDD ratio = 3) will have a time to PZT almost twice as long as the eggs inside a platform nest (19 minutes vs. 11 minutes, respectively). Typically, spotted owl females leave the nest for 12–15 minutes, with most trips lasting <15 minutes [24], [48]. Assuming our calculations of time to PZT were accurate, and female spotted owls maintained their eggs’ temperature above PZT, then a female using the top-cavity nest would not have to constrain her foraging trips. Conversely, a female using the platform nest may have to constrain her time away from the nest during severe weather conditions.

By selecting more insulative nests, incubating females may also decrease their energetic expenditure. In a review of incubation energetics by Thomson et al. [49], metabolic rates of birds incubating outside of their thermo-neutral zone were on average 1.6 times their resting metabolic rate (RMR), but for individuals incubating within their thermo-neutral zone, incubation did not elevate their metabolic rate above RMR. Thus, if a nest minimizes convective heat loss, a female will experience a more favorable microclimate and reduce the energy needed to stay warm and heat the eggs. If a female is required to use less energy to maintain her body temperature and the eggs’ temperature, she will be able to incubate for longer periods before having to forage on her own if her mate does not provide food. The stability of the nest microclimate over time is also important for egg development. The periodic cooling of eggs requires a disproportionate increase in the total amount of energy needed for eggs to fully develop [30]. Eggs that experience periodic cooling also develop more slowly and less efficiently, and young hatched under such conditions are comparatively smaller than young hatched from eggs that develop under constant temperature [30], [50], [51], which has consequences for the ultimate fitness of young developing under these conditions.

Given that top-cavity nests provide optimal thermal environments, silvicultural treatments in forests inhabited by spotted owls should retain older, legacy trees with deformities such as broken tops because these trees may more likely develop into suitable nest trees. Recent climate models suggest that the Pacific Northwest will experience warmer, wetter winters with a 5% increase in precipitation during the nesting season of spotted owls in the next 30 years [52]. Given that spotted owl reproduction is negatively affected by large amounts of precipitation [22], [53], [54], increasing the number of cavity type nests, especially in areas of younger forest, may help counter the potential negative effects of climate change. Cavity type nests are also more stable over time; Folliard [17] stated that platform nests are ephemeral in nature, while cavity nests have annual survival rates approaching 0.992 [18]. Thus, cavity nests provide spotted owls with a thermally optimal environment under a variable climate regime, which is maintained over a long period of time as a semi-permanent component of their habitat.

## Supporting Information

Table S1 **Ranking of** ***a priori*** **models with** ***w_i_*** **>0.001 based on AIC_c_ values.** Acronyms for variables are defined in Table 1.
(DOCX)

Table S2 **Ranking of final** ***post hoc*** **model relative to top 2** ***a priori*** **models from Table S1.** Acronyms for variables are defined in Table 1.
(DOCX)

Table S3 **Parameter estimates, standard errors (SE), and 95% CI for the** ***post hoc*** **model chosen as the best approximating model.** Acronyms for variables are defined in Table 1.
(DOCX)
