# Supplementary material for: Potential Influences of Climate and Nest Structure on Spotted Owl Reproductive Success: A Biophysical Approach
Source: PLoS One. 2012 Jul 31;7(7):e41498. doi: 10.1371/journal.pone.0041498 (PMC3409232; doi:10.1371/journal.pone.0041498)
Supplement: Table S1 — Ranking of a priori models with wi >0.001 based on AICc values. Acronyms for variables are defined in Table 1. (DOCX) [file pone.0041498.s001.docx]

**Table S1.**

| ***a priori* model** | **-2ln*L*** | **K** | **AIC_c_** | **ΔAIC_c_** | ***w_i_*** |
| --- | --- | --- | --- | --- | --- |
| 1) DEPTH + BL + log_e_(RE) + DEPTH x BL + BL x log_e_(RE) | -197.11 | 16 | -158.48 | 0.00 | 0.729 |
| 2) DEPTH + BL + log_e_(RE) + DEPTH x BL | -184.09 | 12 | -156.46 | 2.02 | 0.266 |
| 3) DEPTH + BL + RE + DEPTH x BL + BL x RE | -186.32 | 16 | -147.68 | 10.80 | 0.003 |
| 4) DEPTH + BL + RE + DEPTH x BL | -174.14 | 12 | -146.51 | 11.97 | 0.002 |
