# Supplementary material for: Potential Influences of Climate and Nest Structure on Spotted Owl Reproductive Success: A Biophysical Approach
Source: PLoS One. 2012 Jul 31;7(7):e41498. doi: 10.1371/journal.pone.0041498 (PMC3409232; doi:10.1371/journal.pone.0041498)
Supplement: Table S2 — Ranking of final post hoc model relative to top 2 a priori models from Table S1. Acronyms for variables are defined in Table 1. (DOCX) [file pone.0041498.s002.docx]

**Table S2.**

| **Model** | **-2ln*L*** | **K** | **AIC_c_** | **ΔAIC_c_** | ***w_i_*** |
| --- | --- | --- | --- | --- | --- |
| 1) *Post hoc* model | -193.81 | 13 | -163.53 | 0.00 | 0.901 |
| 2) DEPTH + BL + log_e_(RE) +  DEPTH x BL + BL x log_e_(RE) | -197.11 | 16 | -158.48 | 5.05 | 0.072 |
| 3) DEPTH + BL + log_e_(RE) + DEPTH x BL | -184.09 | 12 | -156.46 | 7.07 | 0.026 |
