# Supplementary material for: Potential Influences of Climate and Nest Structure on Spotted Owl Reproductive Success: A Biophysical Approach
Source: PLoS One. 2012 Jul 31;7(7):e41498. doi: 10.1371/journal.pone.0041498 (PMC3409232; doi:10.1371/journal.pone.0041498)
Supplement: Table S3 — Parameter estimates, standard errors (SE), and 95% CI for the post hoc model chosen as the best approximating model. Acronyms for variables are defined in Table 1. (DOCX) [file pone.0041498.s003.docx]

**Table S3.**

| **Parameter** | **Estimate** | **SE** | **95% CI** |
| --- | --- | --- | --- |
| Intercept | -10.788 | 0.304 | -11.384, -10.193 |
| DEPTH | -0.020 | 0.001 | -0.022, -0.018 |
| BL 0° | 0.347 | 0.046 | 0.256, 0.438 |
| BL 60° | 1.002 | 0.538 | -0.053, 2.057 |
| BL 120° | 0.221 | 0.040 | 0.142, 0.299 |
| BL 180° | 1.787 | 0.642 | 0.528, 3.045 |
| Log_e_(RE) | 0.817 | 0.026 | 0.766, 0.867 |
| DEPTH x BL0 | -0.017 | 0.002 | -0.021, -0.013 |
| DEPTH x BL60 | 0.005 | 0.002 | 0.002, 0.008 |
| DEPTH x BL120180 | 0.009 | 0.002 | 0.006, 0.012 |
| BL60 x Log_e_(RE) | -0.120 | 0.046 | -0.210, -0.030 |
| BL180 x Log_e_(RE) | -0.135 | 0.054 | -0.241, -0.029 |
